# Diversity of nectar amino acids in the *Fritillaria* (Liliaceae) genus: ecological and evolutionary implications

**DOI:** 10.1038/s41598-019-51170-4

**Published:** 2019-10-23

**Authors:** Katarzyna Roguz, Andrzej Bajguz, Magdalena Chmur, Agnieszka Gołębiewska, Agata Roguz, Marcin Zych

**Affiliations:** 1 Botanic Garden, Faculty of Biology, University of Warsaw, Warsaw, Poland; 2 Department of Plant Biochemistry and Toxicology, Institute of Biology, Faculty of Biology and Chemistry, University of Bialystok, Bialystok, Poland; 3 Feature Forest, Trzy Lipy 3, 80-172 Gdańsk, Poland

**Keywords:** Evolutionary ecology, Ecosystem services

## Abstract

Nectar is considered to be a primary food reward for most pollinators. It mostly contains sugars, but also has amino acids. The significance of the concentration and composition of amino acids in nectar is often less understood than that of its volume, sugar concentration and composition. However, there is a trend towards a broader approach in ecological research, which helps to understand nectar properties in an ecological context. The genus *Fritillaria*, exhibiting great diversity in flower morphology, nectar composition, and dominant pollinators, allows for the possibility to study some of the above. We studied the concentration and composition of amino acids in the nectar of 38 *Fritillaria* species attracting different groups of pollen vectors (bees, flies, passerines, and hummingbirds). The flowers of fritillaries produced nectar with a varying composition and concentration of amino acids. These differences were mostly associated with the pollinator type. The nectar of passerine bird-pollinated species was rich in amino acids, whereas humming bird-pollinated produced low amino acid nectar. Contrary to previous reports nectar of the insect-pollinated species did not contain a higher amount of proline. Two non-protein amino acids, sarcosine and norvaline, were detected in the floral nectar for the first time.

## Introduction

Nectar is the most crucial floral reward for animal pollinators^1^. Primarily, it is a sugar solution composed of sucrose, fructose and glucose, in varying proportions. Nectar may also contain other sugars, for example mannose, maltose, and melezitose, as well as trace quantities of many other chemical compounds, including amino acids (AAs)^2–5^.

While nectar sugars generally represent the nectar’s energetic value^1,2^, and their proportion seems to be conserved within a species^2,4,6^, the biological functions of nectar AAs may vary^2,7,8^. The concentration of nectar AAs is traditionally perceived as an adaptation to various pollinator types. For example, plants pollinated by bees and hummingbirds contain a lower AA concentration, whereas butterfly or passerine bird-pollinated flowers produce nectar that is rich in AAs^9,10^. Floral nectar, for example for insects, serves as a dietary source of essential amino acids (EAAs), which are crucial for growth, somatic maintenance, and reproduction^11,12^. A recent analysis of functional flower trait diversity showed that among various floral characteristics, the concentration of nectar AAs is one of the most important traits shaping plant-pollinator interactions^13^. This may be due to the fact that as an essential source of nitrogen for mutualists nectar AAs^4^ also contribute to the taste of the nectar and thus stimulate the chemosensory receptors of insects^2^. Furthermore, compounds such as proline are sources of short-term energy bursts and can be important in the first phases of insect flight^14,15^. Others, such as GABA (γ-aminobutyric acid), taurine and β-alanine, appear to influence insect behaviour by limiting excessive states of excitation during stressful conditions^16^, or induce higer level of locomotion^17^. Also nonessential amino acids (NEAAs) may play an important role, incorporated from the adult diet into the eggs of butterflies may improve reproductive success of butterflies under suboptimal larval conditions^11^.

The role played by nectar AAs extends beyond plant-pollinator interactions. For example, GABA, a non-protein AA (NPAA), may contribute to the protection of nectar from invasion by pathogenic organisms^8^. Moreover, in contrast to the idea of the species-specific constancy of nectar AAs^9^, recent studies show a considerable variability of AA composition within a species or among closely related taxa^18–22^. The above arguments demonstrate that floral nectar is much more than a simple food reward for animals. It is under pollinator-mediated selection and should rather be regarded as a complicated multifunctional interface between plants, their mutualists, and antagonists^3,5,8^.

Unfortunately, non-sugar constituents of nectar are relatively understudied. This is partly because they constitute a small fraction of nectar, as well as exhibit methodological difficulties^8^. Moreover, little is known about phylogenetic constraints on nectar production. To the best of our knowledge only a handful of studies addressed the issue of the nectar AA profile of closely related taxa^20,21^. To that end, we undertook a broad systematic survey of nectar AAs for 38 species of the monocotyledonous genus *Fritillaria* L. (Liliaceae). It comprised of 100–140 species scattered in the Northern Hemisphere, with a substantial representation concentrated in the Mediterranean region, especially in Turkey, Greece, Iran, and Western North America^23^.

Some members of the *Fritillaria* genus have been previously surveyed for nectar diversity^24–26^. These studies, however, only examined nectar sugars in selected members of the genus. Based on sugar nectar profiles, Rix and Rast^24^ concluded that most *Fritillaria* species are putatively pollinated by bees and wasps. Indeed, floral visitors to *Fritillaria* flowers include Hymenoptera (mostly various species of bees and wasps), as well as many other taxonomic groups of insects, for example, Diptera, Lepidoptera, and Coleoptera^27–31^. Asiatic *F. imperialis* and some North American species (*F. gentneri* and *F. recurva*) appear to be predominantly bird-pollinated^32–35^. This, however, does not necessarily reflect the complete spectrum of pollination systems, since extensive studies of pollination biology have only been carried out on two *Fritillaria* species; namely *F. imperialis* and *F. meleagris*^23,31,32^. Furthermore, little information is available on the non-sugar nectar constituents of *Fritillaria*. Specifically, data on the AA profile is restricted to a single species, namely the *F. graeca*^36^. Our aim, therefore, was to explore the nectar diversity of a large sample of the *Fritillaria* species, representing various infrageneric taxa, as well as their geographic regions and pollination systems.

## Results

The nectar of all the studied species contained AAs. Thirty AA compounds or groups of AAs compounds in varying proportions were found in the floral nectar of studied fritillaries (Table 1). On average 23 different AAs were present in a single nectar sample.

**Table 1:** Composition of amino acids (pmol/µL) in subgenera *Fritillaria*, *Japonica, Korolkowia, Liliorhiza, Petilium, Rhinopetalum, Theresia* and other species; ASP – Asparagine, GLU - Glutamic acid, ASN – Asparagine, SER – Serine, GLN – Glutamine, OSER + HIS – O-Serine + Histidine, GLY – Glycine, THR – Threonine, CIT – Citrulline, ARG – Arginine, BALA – β-Alanine, ALA – Alanine, TAU – Taurine, GABA - Gamma*-*Aminobutyric Acid, BABA - β-Aminobutyric acid, TYR – Tyrosine, AABA - α-Aminobutyric acid, CY2 – Cystine, VAL – Valine, MET – Methionine, NVA – Norvaline, TRP – Tryptophan, PHE – Phenylalanine, ILE – Isoleucine, ORN – Ornithine, LEU – Leucine, LYS – Lysine, HYP – Hydroxyproline, SAR – Sarcosine, PHE – Phenylalanine.

	ASP	GLU	ASN	SER	GLN	OSER + HIS	GLY	THR	CIT	ARG	BALA	ALA	TAU	GABA	BABA	TYR	AABA	CY2	VAL	MET	NVA	TRP	PHE	ILE	ORN	LEU	LYS	HYP	SAR	PRO
***Subgenus Fritillaria***																														
*F.acmopetala*	56	96	55	113	348	13	152	135	187	20	17	97	15	19	0	40	6	5	312	0	4	4	39	148	16	39	5	6	56	6359
*F.amana*	132	399	77	140	95	180	264	244	19	24	10	282	0	22	77	0	52	9	188	34	0	11	325	75	95	0	19	3	18	7
*F.carica*	45	126	29	117	21	9	58	30	0	12	0	109	0	0	0	17	0	0	38	0	0	42	20	23	11	25	7	5	95	48
*F.crassifolia*	27	67	7	28	20	9	9	0	0	17	0	34	0	0	0	12	14	0	20	0	0	0	7	10	10	0	7	1	273	0
*F.drenovskii*	43	59	8	43	150	3	195	9	12	17	0	65	21	11	0	13	2	0	13	0	0	0	26	0	0	19	0	6	61	56
*F.ehrhartii*	60	553	76	17	348	9	0	46	0	61	0	57	0	25	0	14	30	0	49	0	0	43	32	18	18	18	0	33	435	84
*F.elwesii*	369	585	4512	550	2646	54	121	389	50	63	42	383	13	14	0	220	19	7	615	0	0	27	548	174	16	85	16	5	28	2419
*F.gracilis*	283	1770	238	101	3412	46	30	117	44	14	24	101	0	34	0	21	0	0	315	76	0	0	158	214	36	0	0	31	60	210
*F.graeca*	20	71	24	35	228	13	24	16	6	10	0	29	0	17	0	8	3	4	25	0	4	0	11	13	9	0	0	3	124	0
*F.kotschyana*	155	2888	94	278	2371	577	40	410	0	0	30	246	0	693	36	12	5	0	488	14	239	22	202	0	63	0	12	0	0	20
*F. lusitanica*	25	99	10	26	56	4	0	13	14	7	0	25	0	2	0	5	0	3	18	6	0	6	6	9	7	8	0	15	136	
*F.meleagris*	216	472	137	374	6581	46	0	0	30	88	0	479	63	77	0	48	0	35	358	63	0	31	165	298	0	155	11	16	82	173
*F.meleagroides*	210	728	35	281	133	17	643	61	32	37	4	348	61	77	0	30	0	30	129	246	10	31	26	43	4	41	4	11	86	140
*F.michailovskyi*	159	223	26	170	370	14	885	44	30	32	2	252	99	29	0	25	1	28	217	213	3	9	39	156	3	105	14	16	477	185
*F.pallidiflora*	27	37	36	37	112	5	134	18	6	21	5	58	15	40	13	8	5	13	17	3	11	0	10	7	7	10	6	5	40	30
*F.pontica*	107	486	282	566	2040	1362	497	521	56	36	49	417	52	26	0	33	0	17	440	24	11	0	117	232	26	64	5	10	187	103
*F.pyrenaica*	56	51	23	146	145	16	96	31	0	53	0	83	0	175	0	0	0	39	952	0	36	60	0	17	25	0	127	16	102	48
*F.stribrnyi*	248	410	30	75	822	8	26	80	0	11	0	70	0	10	0	30	0	0	182	0	0	22	84	92	0	43	4	7	93	17
*F.thessala*	41	83	89	385	964	1916	78	0	36	18	27	112	0	26	436	0	0	11	333	21	0	29	27	278	48	0	0	5	132	87
*F.tubiformis*	222	852	552	1573	9621	98	182	595	6	44	96	656	0	123	0	43	21	0	1063	24	23	36	312	380	0	248	17	5	32	163
*F.ussuriensis*	79	844	146	17	788	515	0	0	13	3	5	28	0	26	0	15	0	0	550	0	0	15	104	211	0	38	2	19	35	12
*F. uvavulpis*	126	1561	47	313	34	51	256	37	0	9	0	509	0	63	23	0	12	117	364	14	12	150	77	0	147	0	20	22	54	37
*F. verticillata*	16	8	0	36	48	2	14	7	0	6	0	24	0	0	6	0	0	2	10	3	3	7	6	4	7	0	0	2	422	116
*F.whittallii*	46	154	35	49	641	34	15	32	0	0	0	103	0	0	0	20	0	5	72	0	0	22	52	1	18	4	6	1	82	11
***Subgenus Japonica***																														
*F.amabilis*	146	278	1112	138	1145	37	16	123	8	28	27	69	32	14	0	73	115	0	304	0	11	19	102	112	0	23	0	1	5	748
***Subgenus Korolkowia***																														
*F.sewerzowii*	87	166	45	100	824	6	465	24	24	31	17	172	53	43	32	24	0	22	43	5	41	0	22	18	15	54	0	8	61	196
***Subgenus Liliorhiza***																														
*F.affinis*	299	402	32	337	473	23	1817	103	41	67	0	560	106	42	0	38	16	39	70	0	0	0	53	37	27	106	0	70	625	336
*F. camtschatcensis*	298	322	18	378	437	17	1804	40	34	50	0	511	106	26	0	34	0	37	53	0	0	0	29	27	0	86	0	32	860	336
*F.eastwoodiae*	49	105	17	53	79	23	42	49	7	8	2	54	0	0	0	16	8	2	44	6	0	7	49	17	0	23	0	1	18	
*F.gentneri*	39	100	19	42	209	2	196	15	6	10	0	69	20	16	0	9	1	8	20	0	4	3	11	8	2	12	0	6	39	59
*F.recurva*	39	59	4	23	88	0	189	1	4	7	0	65	16	8	0	5	1	6	11	0	0	0	7	4	2	12	0	4	17	39
***Subgenus Petilium***																														
*F. eduardii*	179	368	114	1370	62058	47	348	403	18	13	44	2789	18	12	9	125	21	4	393	61	0	19	504	242	119	369	30	5	21	244
*F.imperialis*	211	1851	118	334	6261	16	272	164	52	18	45	1493	28	42	0	23	0	0	254	0	18	45	70	107	9	81	0	6	56	227
*F.raddeana*	54	196	43	103	719	14	0	70	55	26	0	160	0	9	0	25	0	10	75	0	0	22	40	37	0	44	0	0	50	103
***Subgenus Rhinopetalum***																														
*F.stenanthera*	76	194	0	83	65	25	0	70	16	19	0	215	0	0	0	20	28	11	60	0	0	27	33	51	62	31	0	11	753	119
***Subgenus Theresia***																														
*F.persica*	16	326	25	32	136	5	25	10	0	0	0	143	0	0	0	8	0	6	15	0	0	12	20	7	9	0	6	1	70	0
**Other species**																														
*F.grandiflora*	189	790	1370	1937	7842	3966	444	1152	55	121	201	4347	36	132	0	141	37	82	2390	206	51	275	987	948	0	392	226	105	12	275

In general, all the samples contained one to three dominant AAs, which altogether constituted at least 10% of the AAs per sample. A further two to six different AAs jointly constituted a fraction of 5–10%. The most abundant fraction, regarding the number of AAs, included 14–27 AAs and contributed <5% towards the total. We detected a single dominant AA in the nectars of *F. acmopetala*, *F. eduardii*, *F. meleagris*, namely proline (76%) and glutamine (89% and 66%), respectively for each species. In these cases, the fraction in the range of 5–10% was not detected (Supplementary materials: Table 1).

Glutamine was the most abundant AA in the majority of the studied samples (the mean value for all the samples was 4679 ± 14348 pmol/µL), it had the highest nectar share of all the studied species – on average 22.5% ± 16.6 in all the studied samples. The EAAs needed by honeybees (methionine, tryptophan, arginine, lysine, histidine, phenylalanine, isoleucine, threonine, leucine, valine) were present in the nectar of all the studied species. The percentage of EAAs in the studied species varied between 2% and 51%, with a mean value of 14%. In case of insect-pollinated species, the range lay between 5–51% (mean 16%), for hummingbird-pollinated taxa 6–19% (mean 10%), while in passerine-pollinated 2–6% (mean 4%). Valine was the most common EAA present in 36 samples, followed by threonine (present in seven samples), phenylalanine (present in six samples), leucine (present in three samples), and methionine (present in one sample). The NEAAs (alanine, asparagine, glutamic acid, glutamine, glycine, proline, serine) were also present in all species. The percentage of NEAAs in the studied species varied between 24% and 67%, with a mean value of 59%. In case of insect-pollinated species, the range lay between 2–92% (mean 55%), for hummingbird-pollinated taxa 46–76% (mean 67%), while in passerine-pollinated 82–96% (mean 88%). The NEAAs were present in almost all samples, with the exception of glycine and proline, which were absent in the nectar of five studied species. (Supplementary Materials: Table 1).

BABA (β-Aminobutyric acid) was the rarest AA (the mean value for all the samples was 14.1 ± 63 pmol/µL), found in only nine species, and always present in very low concentrations (with a mean percentage value below 1% of the total concentration).

The species with the highest mean concentration of AAs was *F. eduardii* (62058 pmol/µL). The concentration of its AAs was more than 282 times higher than in *F. pallidiflora* (220.4 pmol/µL), the species with the lowest AAs concentration.

Both non-protein (NPAAs) and protein AAs (PAAs) were detected in all samples. The mean proportion of PAAs and NPAAs for all the studied samples was 21:4, and in 50 out of 53 samples the concentration of PAAs was higher. In *F. eduardii* and *F. imperialis* the PAAs usually constituted more than 99% of the total AAs. For *F. crassifolia* and *F. thunbergii*, the quantity of NPAAs was slightly higher than 50%. O-serine + histidine was the most common NPAAs among all the studied species. Two NPAAs, sarcosine and norvaline, were detected in the floral nectar for the first time.

We recorded significant differences between the concentration and proportion of AAs in the same species (the Wilcoxon signed-rank, p < 0.05) for most of the studied taxa. The differences were not statistically significant (p > 0.05) in *F. recurva*, and *F. acmopetala* (samples from various specimens). Therefore, in the case of these species in further analysis i.e. among species variability the mean values were used.

A comparison of closely related taxa, namely *F. recurva* and *F. affinis*, and their cross *F. gentneri*, also revealed significant differences (p < 0.005) in their concentration of AAs. There were also differences in the composition of AAs between parent plants and the hybrid species (*F. affinis* and *F. recurva* vs. *F. gentneri*).

**Figure 1:**
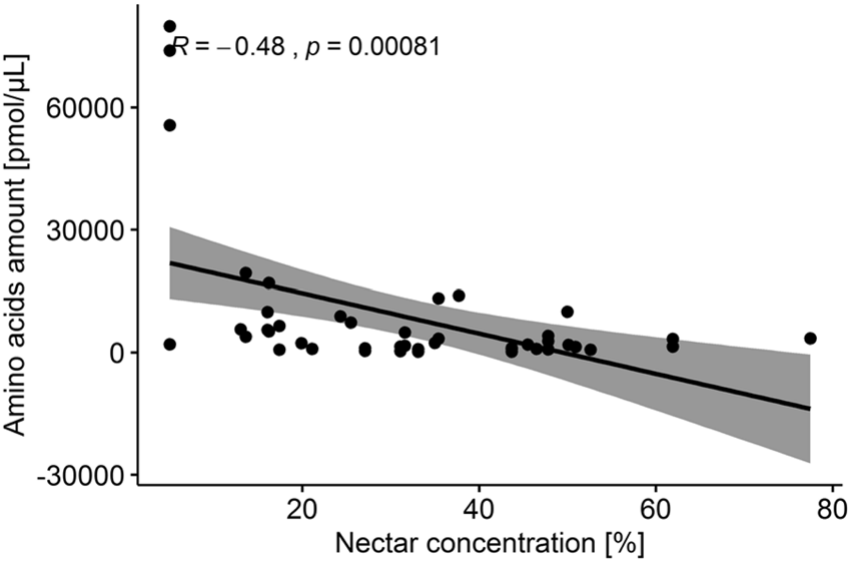
The correlation between the mean concentration of amino acids and nectar concentration (F-statistic: 2.2664, r = −0.48. p = 0.00081), an outlier points are for *F. eduardii*.

Pearson’s correlation revealed negative correlation between sugar and AAs concentration (p < 0.00) and lack of correlation between nectar volume and AAs concentration (Fig. 1). PGLS analysis did not show correlation between nectar properties (sugar concentration and volume, values presented in Table 2) and the total concentration of AAs (P > 0.5).

**Table 2:** Overview of all examined *Fritillaria* species showing OR – origin of the plants (EUR – Europe, AM – North America, AS – Asia), SUB – subgenera (JAP – *Japonica*, FRI – *Fritillaria*, KOR – *Korolkovia*, LIL – *Liliorhiza*, THE – Theresia); some of their main features (v – mean nectar volume produced in one flower, % - mean nectar concentration, MS – main nectar sugar type, FRU, GLU, SUC – amount of different sugar types (mg/ml) following information in Roguz *et al*. 2018), POL – main pollinator type derived from literature data, N/NF/NP – number of samples/number of flowers used for one sample/number of plants used to obtain nectar samples, SOU – source of samples, PIC – corresponding picture.

	OR	SUB	V	%	MS	FRU	GLU	SUC	POL	N/NF/NP	SOU	PIC
*F. acmopetala* Boiss.	EUR	FRI	35	35	FRU	101	30	1	BEE	2/1/2	BG	1
*F. affinis* (Schult. & Schult. f.) Sealy	AM	LIL	15.4	13	FRU	222	144	14	BEE	1/1/1	BG	2
*F. amabilis* Koidzumi	EUR	JAP			FRU	216	118	158	BEE	1/1/1	BG	3
*F. amana* (Rix) R. Wallis & R.B. Wallis	EUR	FRI	8	48	FRU	145	11	364	BEE	1/1/1	BG	4
*F. camtschatcensis* L.	AM/AS	LIL	NA	NA	GLU	24	16	0.6	FLY	1/3/1	BG	5
*F. carica* Rix	EUR	FRI	14	21	FRU:GLU	163	153	25	BEE	1/1/1	BG	6
*F. crassifolia* Boiss. & A. Huet	EUR	FRI			SUC	29	33	55	BEE	1/1/1	BG	7
*F. drenovskii* Degen & Stoj.	EUR	FRI	36	27	GLU	164	189	1	BEE	2/1/2	PK	8
*F. eastwoodiae* R.M. Macfarlane.	AM	LIL	34	16	FRU	98	48	5	BEE	1/1/1	CE	
*F. eduardii* A. Regel ex Regel	AS	PET	57	5	FRU	15	4		PAS	3/1/3	BG	
*F. ehrhartii* Boiss. & Orph	EUR	FRI	9	50	NA	NA	NA	NA	BEE	1/1/1	BG	9
*F. elwesii* Boiss.	EUR	FRI	51	38	FRU	103	39	93	BEE	1/1/1	BG	10
*F. gentneri* Gilkey Madroño	AM	LIL	54	31	FRU	267	155	104	HUM	2/1/2	BG	11
*F. gracilis* Smiley	EUR	FRI	54	26	SUC	36	3	124	BEE	1/1/1	BG	
*F. graeca* Boiss. & Sprun.	EU	FRI	10	53	FRU	98	86	33	FLY	1/1/1	BG	12
*F. grandiflora* Grossh.	EUR	OTH	NA		NA	NA	NA	NA	BEE	1/1/1	BG	
*F. imperialis* L.	AS	PET	205	14	FRU:GLU	33	34	1	PAS	3/1/3	PK	12
*F. kotschyana* Herbert	EUR	FRI	55	24	FRU	97	37		BEE	1/1/1	BG	13
*F. liliacea* Lindl.	AM	LIL	34	48	NA	NA	NA	NA	BEE	2/1/1	CE	14
*F. lusitanica* Wikstr.	EUR	FRI	51	9	SUC	42	5	109	BEE	1/1/1	LH	15
*F. meleagris* L.	EUR	FRI	NA	50	FRU:GLU	NA	NA	NA	BEE	1/1/1	BG	16
*F. meleagroides* Patrin ex Schult. f	EUR	FRI	39	32	FRU	146	34	64	BEE	2/1/2	PK	17
*F. michailovskyi* Fomin	EUR	FRI	4	17	FRU	76	31	55	BEE	2/1/2	BG	18
*F. pallidiflora* Schrenk	AS	FRI	26	44	GLU	147	158	72	BEE	3/1/3	BG	19
*F. persica* L.	EUR	THE	4	47	GLU	215	570	41	BEE	1/1/1	BG	20
*F. pontica* Wahlenb.	EU	FRI	6	16	FRU	54	28	9	BEE	2/1/2	BG	21
*F. pyrenaica* L.	EU	FRI	52	20	SUC	74	29	98	FLY	1/1/1	BG	22
*F. raddeana* Regel.	AS	PET	9	50	SUC	99	69	119	BEE	1/1/1	BG	23
*F. recurva* Benth.	AM	LIL	49	33	FRU	95	54	15	HUM	3/1/3	BG	
*F. sewerzowii* Regel.	EUR	KOR	25	62	FRU:GLU	140	140		BEE	2/1/2	BG	24
*F. stenanthera* (Regel) Regel	AS	THE	1	46	GLU	70	139	10	BEE	1/1/1	BG	25
*F. stribrnyi* Velen.	EU	FRI	7	35	NA	NA	NA	NA	BEE	1/1/1	BG	26
*F. thessala* (Boiss.) Kamari	EUR	FRI	14	16	SUC	40	5	89	BEE	1/1/1	BG	27
*F. tubiformis* Gren. & Godr.	EUR	FRI	1	46	SUC	57	34	72	BEE	1/1/1	BG	
*F. ussuriensis* Maxim.	EUR	FRI	3	78	SUC	87	7	283	FLY	1/1/1	BG	28
*F. uva vulpis* Rix	EUR	FRI	12	48	GLU	90	99	53	BEE	2/1/2	BG	30
*F. verticillata* Willd.	AS	FRI	1	17	GLU	374	407	1	BEE	1/1/1	BG	31
*F. whittallii* Baker	EUR	FRI	14	51	FRU	186	52	167	BEE	1/1/1	BG	32

**Figure 2:**
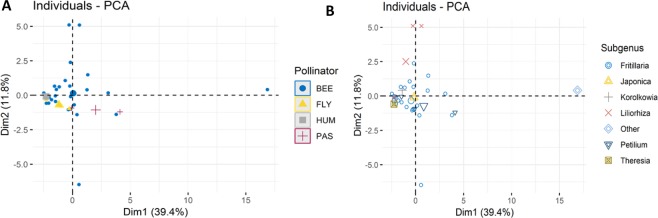
Scatterplot of PCA, (**A**) – data grouped by pollinator, (**B**) – data grouped by subgenus identity.

The first two principal components of the AAs concentration, with the main pollinator and subgenera as explanatory variables, explained 69.2% and 60% (Fig. 2AB) of the total variance, respectively. A PERMANOVA was performed on the AAs composition. Pollinators, subgenera and main sugar were used as categorical variables to complement the graphics evaluation derived by PCA. They were highly significant (p < 0.05) between studied sections, with the exception of the main sugar type (p = 0.099) (Table 3).

**Table 3:** Results of the PERMANOVA: Degrees of freedom (Df). pseudo-F(F). R^2^ and p value.

PERMANOVA	Df	F	R^2^	*p*
**AAs**				
Pollinators	5	2.85	0.23	0.001***
Subgenera	6	1.98	0.21	0.001***
Main sugar	3	1.4	0.09	0.099
Origin	4	1.47	0.11	0.06

Random forest analysis revealed a strong influence of phylogenetic affinity on the composition and concentration of AAs, resulting in 20 out of 25 samples correctly assigned to proper subgenus (with a class error value of 32.26%). The main pollinators type resulted in 23 out of 31 samples correctly assigned to proper subgenus (with a class error value of 25.8%). The variation in the profile of AAs was not explained by origin and the main sugar component of nectar (these saw class errors, respectively of 58% and 51.9%).

The phylogenetic signal was not present (λ ≪ 1).

## Discussion

The mean number of different AAs found in *Fritillaria* nectar (2323), corresponds well with a similar analysis performed on other taxa^22,36–38^. All samples of fritillaries analyzed here contained NPAAs, while Baker and Baker^10^ reported NPAAs to be present in only 36% of the samples, which took part in their extensive study. Such a discrepancy may be related to the applied techniques (the dansylation-UV fluorescence method vs. HPLC), alternatively it might be due to species-specific differences. Two NPAAs, sarcosine and norvaline, were detected in the floral nectar for the first time. A minimum of two and a maximum of nine NPAAs were found in the nectar of fritillaries. This resembles the results obtained for other species from other genera and families studied previously^22,36–39^. The mean concentration of AAs identified in the *Fritillaria* nectar was 8633 ± 16776 pmol/µL and proved to be relatively high when compared to species from other genera and families cited above.

Our study revealed that the total concentration and composition of AAs varies widely within and between the *Fritillaria* species. While a variation in the total concentration of AAs has been previously reported^9,22,39^, the variability in composition is rather unexpected, especially in the case of specimens derived from the same location. There is only one study on supergeneralistic species *Angelica sylvestris* L. showing differences in nectar AAs composition within a species. In this case, however, samples were collected along an ~700-km transect, and the differences in AAs composition could be a result of “adaptive wandering” rather than related to pollination ecotypes adapted to local pollinator assemblages^22^. The mean value of the correlation between AAs composition from nectar samples of the same species, showing infraspecific variability, was r = 0.6 (88% of significant correlations), and was lower compared to the results presented by Gardener and Gillman^39^: namely r = 0.88 (all 544 obtained correlations were statistically significant). The low value of this factor, obtained in our study, may indicate that the species-specific AA composition is not fixed, and might be modulated by environmental factors rather than being determined by genetics alone^18,40^.

Our results are in contrast to the work of Lohaus and Schwerdtfeger^41^ who hypothesised that the avoidance of excessively high nitrogen loss by plants producing large nectar volumes might be achieved by a reduction in the AAs concentration. Both *Petilium* species, *F. imperialis* and *F. eduardii*, produce the highest nectar volumes of all the studied *Fritillaria* species^26^, which also has a high AA concentration.

According to Baker and Baker^42^, in case of hybridization the hybrid nectar is an ‘additive’, which means it contains each of the AAs of the parents, but does not contain any AAs that are not present in the parents’ nectar. The nectar components of *F. gentneri* (a cross between *F. recurva* and *F. affinis*) may be regarded as an ‘additive’ only to some extent. There are two novel AAs not present in either of the parents’ nectar. Moreover, few AAs present in both parental nectars are absent in the nectar of some hybrid flowers. The differences might be related to the fact that Baker and Baker’s^42^ plants were F1 hybrids, while *F. gentneri* is probably a well-established hybrid capable of sexual reproduction^34^. Detected differences might thus indicate an adaptation to pollinators, since *F. recurva* and *F. gentneri* are visited (and probably pollinated) by hummingbirds^34^, whereas *F. affinis* is bee-pollinated. Furthermore, the lack of ‘additiveness’ and the appearance of ‘new’ AAs means that AAs profiles are not predictive of *Fritillaria* phylogeny, and contrary to Baker and Baker^42^ environmental influence is more significant than genetic control. This aspect of nectar biology, however, deserves further attention.

Baker and Baker’s^42^ study discovered that AAs are universally present and follow a species-specific pattern. Similarly to carbohydrates, nectar AAs can play an important role in the attraction of pollinators^6,10,20,43,44^, as they constitute an important nitrogen source for several animal groups^20,45^. The adaptive significance of floral nectar (and its AAs composition) is reflected by its ability to attract potential pollinating agents^20,46,47^. However, pollinators searching for nectar would usually probe multiple flowers^19^ and the nectar composition variability between individuals of one species may be perceived as random noise^19,48^. Therefore, even in the case of pollinators’ preference for a particular nectar composition, the selective pressure on a favoured floral nectar type would be hampered by the high variation within co-flowering individuals^19^.

Nevertheless, the influence of potential pollinators should not be neglected as their flower choice seems to be a key element in the debate concerning the ecological role of AAs in floral nectar^49^. Results from previous studies demonstrate the prevailing importance of direct reward measurements, indicating that AAs concentration is one of the most important traits to shape plant-insect interaction^13^. One of the studies of AAs composition – of floral nectars in a phryganic community – revealed that flower-visiting insects were the most decisive players in shaping nectar chemical composition^36^. This might be related to the fact that insects are a group of pollinators that strongly rely on a flowers’ food source, which results in a strong coevolution of nectar and insect-pollinators^6,39^. This seems to be the case in *Fritillaria* pollinated by different groups of animals. Our studies revealed substantial differences in the nectar composition of fritillaries, even among closely related species. This suggests an adaptation to potential pollinators. These differences relate not only to AA compounds but also to carbohydrates^26^.

Although data concerning pollination in *Fritillaria* is scarce, nectar properties and nectary location indicate that bees are most likely the commonest pollinator of *Fritillaria* flowers^26^. Therefore, we expected the nectar of fritillaries to be rich in proline, which is oxidized in the insects’ flight muscle, especially during the first phase of flight^15^. Moreover, proline is a type of AA, which accumulates at a high concentration in nectars of many angiosperm species^2,4,7,50,51^, regardless of their phylogenetic distances^8,51^. Since insects have the ability to taste proline and favour proline-rich nectar, they are likely to impose selective pressure on plant species producing proline-containing nectars^14^. Surprisingly, in the case of fritillaries proline was the most abundant AA in only a single insect-pollinated species, namely *F. acmopetala*, and the share of this AA in the nectar of other *Fritillaria* was relatively low. If present, the quantity of proline in the nectar of insect-pollinated species was variable and ranged from 7 to 6359 pmol/µL. Such low concentration of proline may result from the fact that proline is metabolically more expensive than other nectar compounds^14^. Phylogenetic constraints may also play a role for some subgenera, as some closely related species have a similar concentration of proline. These include *F. verticillata* and *F. pallidiflora*. Phenylalanine, which is also one of the EAAs, generates a strong phagostimulatory effect on bees^36^, and was also present in small concentration in the nectar of the bee-pollinated *Fritillaria* species (with a mean ratio of 2%). From the three most abundant AAs in *Fritillaria* nectar i.e., glutamine, glutamic acid, and glycine, only the role of glycine is known. This AA has a strong influence on honey bee-learning behaviour^52^. As for the other two AAs, these seem to be consistent and sizable nectar components of certain species^18,39^. However, the evaluation of their role in plant-pollinator interaction must await further study. We still lack evidence, but the *Fritillaria* reward composition may be related to the pollinator’s life cycle. Fritillaries are spring–flowering plants. They flower almost immediately after the snow melts and are thought to be Queen bee pollinated^23^. The AAs requirements of *Fritillaria* pollinators could thus be very specific, for instance, for reproduction.

We also lack studies on the metabolism and ecological role of NPAAs. However, an ‘ecophysiological’ picture has emerged^8^. Proline is utilized during the first phase of insect flight, while nectar sugars propel long-distance flights, and taurine, GABA and β-alanine increase the efficiency of flight muscles. Moreover, proline and GABA increase the insect’s appetite for nectar due to phagostimulatory activity^7^. Surprisingly, β-alanine is not present in *Fritillaria* nectar, while taurine is present only rarely, and GABA is found in low concentration. We found two NPAAs, sarcosine and norvaline, for the first time in floral nectar, but its influence on pollinators is not known.

Some studies have revealed a trade-off between carbohydrate quantity and AAs abundance. This carbohydrates-to-AAs ratio could play a functional role as, for example, it has been demonstrated that honey bees would rather acquire essential AAs than sugars^4,53^. Our study showed a similar trend for *Fritillaria*, but the results were statistically non-significant. Moreover, not all EAAs were detected in the nectar of melittophilous species. However, since pollen seems to represent an additional source of nitrogen and AAs, bees do not appear to solely rely on nectar to supply these substances^21^. In this case the relative abundance of the different AAs (including the essential ones), could play an important role in providing potential pollinators with specific taste-information used in the field for food resource selection^54^.

Similarly to butterfly-pollinated flowers, a high level of AAs was also described in flowers pollinated by carrion flies^3,10,45^. Flesh flies have been known to select nectar containing a mixture of AAs^55^. Six EAAs (i.e. valine, leucine, isoleucine, methionine, phenylalanine, tryptophan) elicited a feeding response by stimulating flies’ chemoreceptors^56^. Flies were reported to visit flowers of *F. camtschatcensis*, however, contrary to previous results obtained by Baker and Baker^10^ for other fly-pollinated species, the relative concentration of AAs in *F. camtschatcensis* nectar was considerably lower than the mean value. Moreover, the six AAs that caused a feeding response in flies were also hardly present in the nectar of this species.

The phylogeny^57^ and field observation of the *Fritillaria* pollination system suggests that there have been at least two transitions from entomophily to passerine or hummingbird pollination^25,26,32,33,58^. These transitions involve several floral modifications, and are accompanied by changes in nectar volume and concentration, as well as sugar composition^26^. Previous studies of Baker and Baker^10^ indicate, that AAs content may also change due to a pollinator shift. *Fritillaria imperialis*^32,33^, and the closely related *F. eduardii*, both likely pollinated by passerine birds^26^, were indeed reported to have a distinct AAs concentration and composition. The total concentration of AAs in the nectar of these two species was higher than the mean value, which is in accordance with several other observations of passerine pollinated species^2,10,21,45^. Such a high AAs concentration in bird-pollinated flowers may also have a repellent character, since a hymenoptera dominated pollinator community avoids high AAs concentration in floral nectar^13^.

Similarly to the passerine-pollinated *Erythrina* species^2^, glutamine, in bird-pollinated *Fritillaria*, occurred in much higher concentrations. Although EAAs were found to be commonly present in passerine-pollinated species^2^, they are virtually absent in the nectar of both *F. imperialis* and *F. eduardii*. NEAAs are the main drivers of the variable concentration in fritillaries. Interestingly, the third member of the subgenus *Petilium*, *F. raddeana*, produced nectar with a lower concentration of AAs and higher percentage of both EAAs and PAAs. All the above-mentioned facts seem to indicate that the non-sugar components of nectar may play an important role in the plant-pollinator interactions^2,59^. A high AAs concentration with low nectar sugar concentration may play the role of a phenotypic filter, deterring illegitimate pollinators and antagonists, since various insects have shown a distaste for a high concentration of AAs. This could be masked by a higher concentration of carbohydrates^4^.

In the case of *F. gentneri* and *F. recurva*, which are both hummingbird-pollinated species, the total AAs concentration was 11 times lower than the mean value counted for all studied species. This dichotomy of AAs concentration in ornithophilous species, with a high concentration in passerine-pollinated species and lower concentrations in hummingbird-pollinated species, has already been described in other taxa^2,10,21,45^, as well as experimental studies^60^. Low AAs concentration in hummingbird-pollinated flowers may also have a repellent effect, aiding avoidance of competition with bees favouring higher AAs concentrations^3,10,45^. On the other hand, the shortage of AAs in a bird’s diet could be overcome via additional food sources, e.g. insects^61^. Similarly to subgenus *Petilium*, we found differences in AAs concentration and composition in the nectar of closely related species in the subgenus *Liliorhiza*. *Fritillaria affinis*, *F. eastwoodiae*, *F. liliacea* produced nectar with a higher AAs concentration. Furthermore, a higher concentration of bee-preferred proline was present in species of this subgenus presumed to be insect-pollinated, which also indicates a strong influence of pollinators.

The influence of colonizing microorganisms on AAs, and their effect on plant-pollinator interaction, is also an important factor^51,62,63^. However, due to difficulties in obtaining nectar samples we could not study this aspect of *Fritillaria*. Nevertheless, we assume that pending flowers of most *Fritillaria* species are a kind of protection from microorganisms transported with the air, and therefore can reduce the number of nectar-inhabiting microorganisms. However, further research is needed before drawing any strong conclusions in this regard. Other interesting aspects for future AAs studies include the impact of non-standard, psychoactive AAs on pollinators^7,64^. Moreover, since the AAs composition in nectar correlates with AAs composition in phloem sap^41^, the next step in studying nectar variability would be to examine to what extent the differences between and within the species are correlated with the composition of phloem sap versus influenced by a pollinator.

AAs concertation and composition in *Fritillaria* may be influenced by several factors. While phylogeny plays a role, as several closely related species have a similar AAs composition and concentration, the prevailing evidence is that AA composition and concentration can be variable even within a single species, as well as between closely related taxa. This is in agreement with the results of Gijbels *et al*.^19^ and Lanza *et al*.^18^, who found differences in the concentration and composition of AAs at the species level. Glutamine, glutamic acid, and glycine were the most abundant AAs in the nectar of the *Fritillaria* species analyzed in this study, but further analysis is needed to assess the potential role of these AAs. However, our results contradict other studies, which suggest that proline is the most abundant AA in floral nectar^9,36,65^.

The nectar traits studied can be subject to selection, which is imposed by potential pollinators, a finding confirmed by other researchers^36,66,67^. Changes in AAs concentration and composition might play an important role in attracting new floral visitors in case of a pollinator shift. Our analysis revealed high concentration of AAs in passerine bird-pollinated species, and very low AAs concentration in hummingbird-pollinated species. These tendencies were not reflected in closely related species from the same subgenus.

## Material and Methods

### Taxon sampling

**Figure 3:**
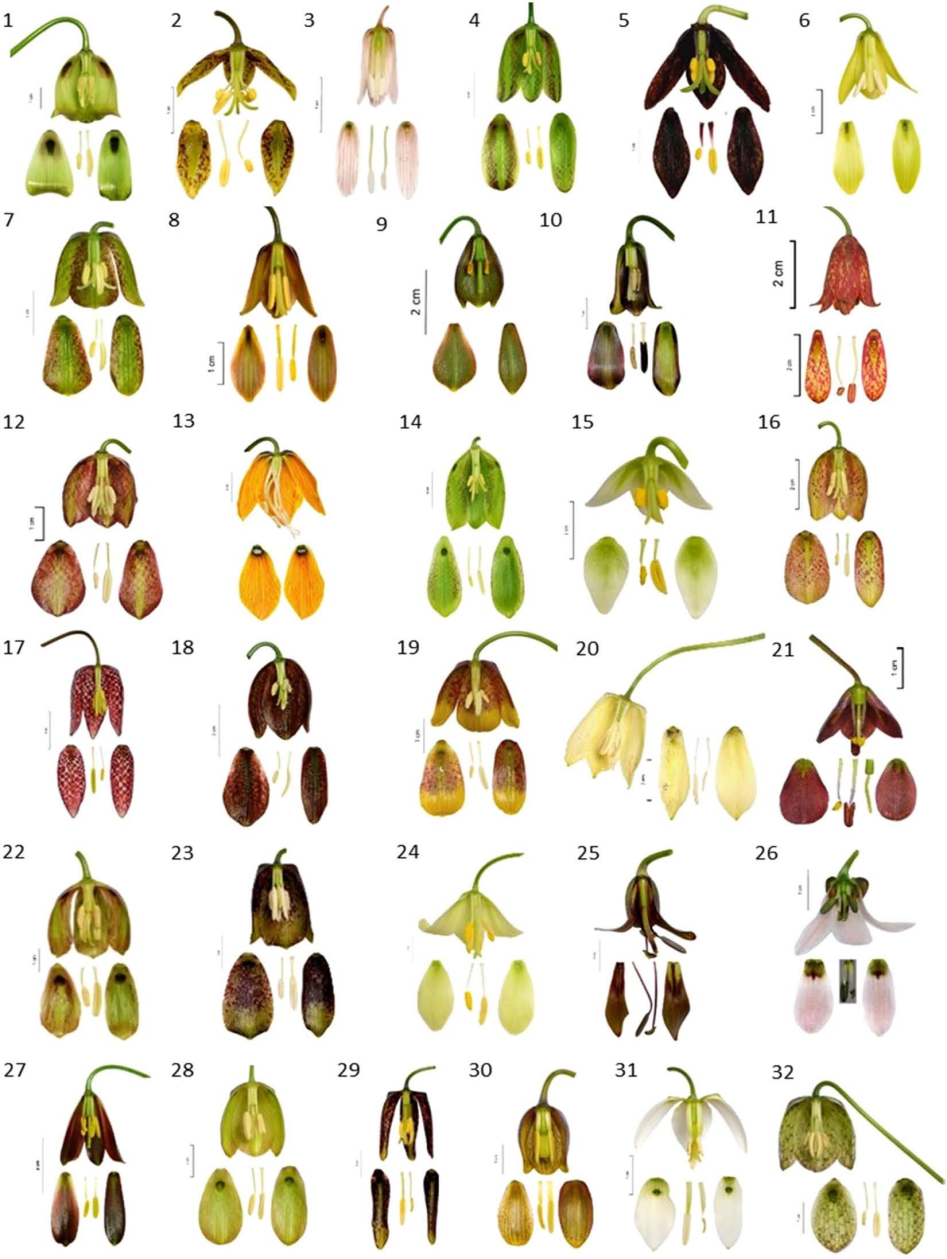
Flowers and nectaries of selected *Fritillaria* species in full anthesis (species’ names in Table 2).

Nectar samples used for this study were obtained from the *Fritillaria* species cultivated at the University of Warsaw Botanic Garden (hereafter BG) and in the private collections of Colin Everett (Somerton, Somerset, UK; hereafter CE), Laurence Hill (Richmond, Surrey, UK; hereafter LH) and Paweł Kalinowski (Szczeglacin, Korczew, Poland; hereafter PK) (Fig. 3). Most of the *Fritillaria* species are rare in cultivation, and the number of specimens used in the study varied due to the availability of flowers or their nectar (the accession numbers for species used in this study are listed in Table 2). Flowers in the collections of BG and PK were first selected at the bud stage (flowers still closed) and bagged with nylon mesh (net size 0.5 mm) to prevent visits by insects. In all cases the nectar was sampled at the anthesis stage before the anthers had dehisced. In case of LH’s and CE’s collections, nectar sampling was performed on unbagged flowers exposed to animal visitors (possible contamination of floral nectar with pollen grains during insects visits). Due to possible influence of potential flower visitors in the case of unbagged flowers, these species were excluded (*F. liliacea, F. eastwoodiae, F. lusitanica*) from analyzis.

All the available nectar was sampled with microcapillary pipettes from the nectaries of all six tepals and treated as one sample per flower. In the case of *F. camtschatcensis*, where nectar volumes were very small, samples were taken under a Nikon SMZ 1000 stereomicroscope (Nikon Corp., Japan). For species producing small quantities of nectar several flowers of the same specimen were used to collect it. Only the *F. liliacea* samples taken from different flowers of a single specimen were considered to be separate samples. During sampling we tried to avoid possible pollen, phloem sap or other plant tissue contamination, since it could affect the obtained results by releasing additional AAs^68–70^. All the nectar samples were collected around noon (between 11am and 1 pm). The collected nectar was frozen at −20 °C in 1.5 ml Eppendorf tubes prior to analysis.

### Nectar AA composition analysis

The composition of the nectar’s AAs was analyzed using high-performance liquid chromatography (HPLC). The samples were frozen (−20 °C) until determination. After thawing the samples to an ambient temperature the nectar was diluted to a volume of 20 μL (10 μL of nectar was mixed with 10 μL of distilled water). The sample was filtered through a spin column with a 0.4 µm pore size membrane filter (A&A Biotechnology, Poland) before injection by centrifugation for 2 min at 9000 g (relative centrifugal force). The supernatant was loaded into the insert and analyzed by a HPLC. The samples were analyzed using an Agilent Technologies 1260 Infinity series system consisting of a 1260 Infinity Agilent Quaternary pump G1311B, a 1260 Infinity Diode Array Detector (DAD) G1315D, a 1260 Infinity Fluorescence Detector (FLD) G1321B, a 1260 Infinity ALS G1329B Automated Sample Injector, a 1290 Infinity Autosampler Thermostat G1330B and a thermostatted column oven 1290 Infinity TCC G1316C. The system was controlled by Agilent OpenLab ChemStation software. The analysis of AAs in 10 μL aliquots of nectar collected from flowers was performed by gradient HPLC using an Agilent Zorbax Eclipse Plus C18 (4.6 × 150 mm, 5 μm) column with a guard, i.e. Agilent Zorbax Eclipse Plus C18 (4.6 × 12.5 mm, 5 μm). The extracts, containing primary and secondary AAs were pre-column derivatized with *o*-phtalaldehyde (OPA) and 9-fluorenylmethyl chloroformate (FMOC) reagent. An injector program was used for the derivatization. Following derivatization, a mixture of each sample was injected into a pre-equilibrated column operated at 40 °C. The primary (OPA-derivatized) AAs were monitored at 388 nm by DAD while the secondary (FMOC-derivatized) AAs were monitored by FLD, at an excitation wavelength of 266 nm and an emission wavelength of 305 nm. Mobile phase A was 40 mM NaH_2_PO_4_ (pH 7.8 adjusted using 10 M NaOH solution), while mobile phase B was acetonitrile:methanol:water (45:45:10. v/v/v). The following gradient profile was seen: 0–5 min: 0% B t- 10% B; 5- 25 min: 10% B - 40.5% B; 25–30 min: 40.5% B - 63% B; 30–35 min: 63% B - 82% B; 35–37 min: 82% B - 100 B; 37–39 min: 100% B; 39–40 min: 100% B- 0% B; 40 43 min: 0% B. A flow rate of 1 mL/min was used. The total analysis time was 43.0 min.

### Data analysis

The total concentration of AAs was determined for each species. If several samples were obtained the results were used in the analyzis as separate records. The percentage of EAAs, non-protein amino acids (NPAAs), and protein amino acids (PAAs) was calculated. The Wilcoxon signed-rank test was applied to determine significant differences between the AAs composition in samples from the same or very closely related species. The correlations between nectar properties (concentration of sugars and volume) were calculated using the Pearson’s product moment correlation coefficient. Additionally, the phylogenetic generalized least squares (PGLS) correlation was calculated, since this approach accounts for independent variables^71^.

A principal component analysis (PCA) was performed and plotted against subgenera and the main pollinator, to visualize the differences in the concentration of AAs. The data on pollinators (see Table 1) was gained from the available literature^27,29–34,72–74^, personal field observations (*F. michailovskyi, F. raddeana, F. carica, F. pontica, F. sewerzowii, F. ussuriensis, F. uva-vulpis F. persica, F. acmopetala, F. pallidiflora*), or based on the morphological adaptations of *Fritillaria* flowers. In the latter case, predictions of the pollinators syndrome were made from the following flower characteristics: corolla shape, size, colour, position on a stem, and nectar sugar composition and concentration^26^.

A permutational multivariate analysis of variance (PERMANOVA) was conducted to identify the relative importance of all the sugar concentrations, pollinators, subgenera, and origin of the species. The Adonis routine was used for this purpose (it offers a multivariate analysis of variance using distance matrices based on the permutation test). ‘Random forest’ analysis was used to test whether nectar AAs differed between subgenera and the pollinators type and origin^75^. This machine-learning algorithm allows the assignment of nectar samples to pre-defined groups of subgenera and a pollinator type and origin. Random forest returned a confusion matrix showing the number of correctly assigned samples for each request.

The maximum likelihood (ML) tree was inferred with the use of the matK sequences obtained from GenBank. The fast bootstrap method implemented by IQ-TREE 1.6.8 was used for ML bootstrap analysis^76^. Lengths of tree branches were obtained from a ML tree of the *Fritillaria* genus. The phylogenetic correlation λ was calculated to assess the phylogenetic signal in the analyzed data. Pagel’s λ is the transformation of the phylogeny, ensuring the best fit of studied trait data to a Brownian Motion model^77,78^.

All the statistical analysis was performed using^79^ (version 3.3.5. www.r-project.org.).

## Supplementary information

Dataset 1

## Data Availability

The datasets generated during and/or analysed during the current study are available from the corresponding author on reasonable request.
