# Author Correction: Diversity of nectar amino acids in the *Fritillaria* (Liliaceae) genus: ecological and evolutionary implications

**DOI:** 10.1038/s41598-020-58145-w

**Published:** 2020-01-21

**Authors:** Katarzyna Roguz, Andrzej Bajguz, Magdalena Chmur, Agnieszka Gołębiewska, Agata Roguz, Marcin Zych

**Affiliations:** 1 Botanic Garden, Faculty of Biology, University of Warsaw, Warsaw, Poland; 2 Department of Plant Biochemistry and Toxicology, Institute of Biology, Faculty of Biology and Chemistry, University of Bialystok, Bialystok, Poland; 3 Feature Forest, Trzy Lipy 3, 80-172 Gdańsk, Poland

Correction to: *Scientific Reports* 10.1038/s41598-019-51170-4, published online 23 October 2019

This Article contains typographical errors in the Acknowledgements section.

“The study was financially supported by research Grant No. 4786 2013/11/N/NZ8/00611 from the Polish National Science Centre (to K.R.), and tests of the HPLC method for AAs analysis were supported via the Polish National Science Centre grant 2011/01/B/NZ8/03146 (to M.Z.).”

should read:

“The study was financially supported by research Grant No. 4786 2013/11/N/NZ8/00611 from the Polish National Science Centre (to K.R.), and tests of the HPLC method for AAs analysis were supported via the Polish National Science Centre grant 2014/15/B/NZ8/00249 (to M.Z.).”

