# Supplementary material for: Diversity of nectar amino acids in the Fritillaria (Liliaceae) genus: ecological and evolutionary implications
Source: Sci Rep. 2019 Oct 23;9:15209. doi: 10.1038/s41598-019-51170-4 (PMC6811550; doi:10.1038/s41598-019-51170-4)
Supplement: Supplementary file 1 — Dataset 1 [file 41598_2019_51170_MOESM1_ESM.docx]

**Diversity of nectar amino acids in the *Fritillaria* (Liliaceae) genus: ecological and evolutionary implications**

Katarzyna Roguz^1^, Andrzej Bajguz^2^, Magdalena Chmur^2^, Agnieszka Gołębiewska^2^, Agata Roguz^3^ and Marcin Zych^1^

^1^Botanic Garden, Faculty of Biology, University of Warsaw, Warsaw, Poland

^2^Department of Plant Biochemistry and Toxicology, Institute of Biology, Faculty of Biology and Chemistry, University of Bialystok, Bialystok, Poland

^3^Feature Forest, Trzy Lipy 3, 80-172 Gdańsk, Poland

**Correspondence:**

**k.roguz@biol.uw.edu.pl**

Supplementary materials:

Maximum-likelihood tree of studied *Fritillaria* genus. constructed with the IQ-TREE platform^46^.


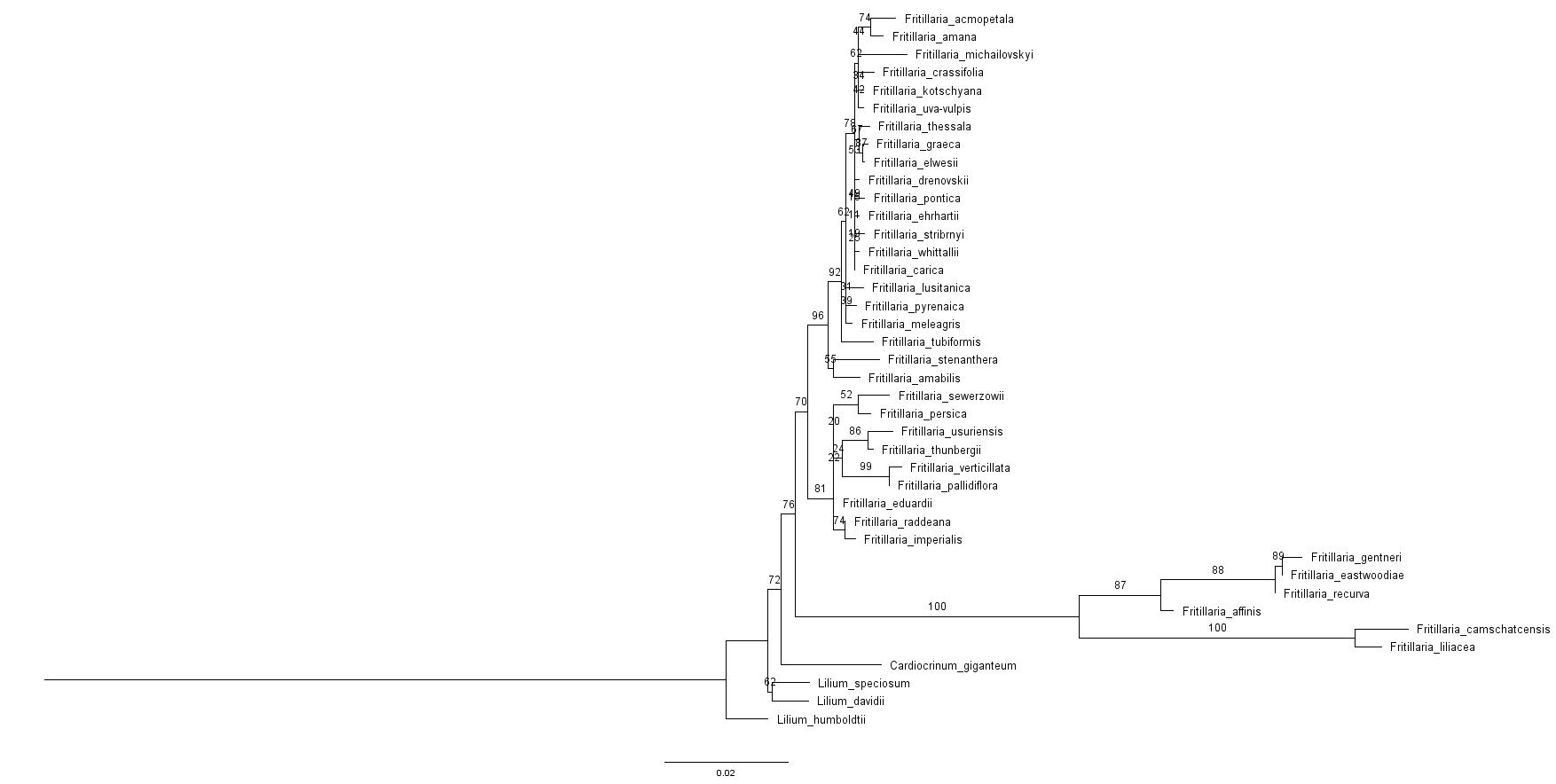


Table 1. Percentage composition of amino acids (AAs) in subgenus *Fritillaria* plants, for the following species: *F. acmopetala,* *F. drenovskii, F. eduardii, f. gentneri, F. imperialis, F. liliacea, F. meleagroides, F. michailovskyi, F. pallidiflora, F. pontica, F. recurva, F. uva vulpis* the mean values are presented. The AAs essential for honeybees are marked with “^”, the AAs nonessential for butterflies are marked with”*”, the no-protein AAs are marked with “#”, ASP – Asparagine, GLU - Glutamic acid, ASN – Asparagine, SER – Serine, GLN – Glutamine, OSER+HIS *-* O-Serine+Histidine*,* GLY – Glycine, THR – Threonine, CIT – Citrulline, ARG – Arginine, BALA – β – Alanine, ALA – Alanine, TAU – Taurine, GABA - Gamma*-*Aminobutyric Acid, BABA - β-Aminobutyric acid, TYR – Tyrosine, AABA - α-Aminobutyric acid, CY2 – Cystine, VAL – Valine, MET – Methionine, NVA – Norvaline, TRP – Tryptophan, PHE – Phenylalanine, ILE – Isoleucine, ORN – Ornithine, LEU – Leucine, LYS – Lysine, HYP – Hydroxyproline, SAR – Sarcosine, PRO – Proline.

|  | ASP* | GLU* | ASN | SER* | GLY | ALA* | VAL^ | OSER+HIS^ | THR^ | CIT# | ARG^ | BALA | GLN | TAU# | GABA# | BABA# | TYR^ | AABA# | CY2# | MET^ | NVA# | TRP^ | PHE^ | ILE^ | ORN# | LEU^ | LYS^# | HYP# | SAR# | PRO# |
| --- | --- | --- | --- | --- | --- | --- | --- | --- | --- | --- | --- | --- | --- | --- | --- | --- | --- | --- | --- | --- | --- | --- | --- | --- | --- | --- | --- | --- | --- | --- |
| *F. acmopetala* | 0.7 | 1.1 | 0.7 | 1.3 | 1.8 | 1.2 | 3.7 | 0.2 | 1.6 | 2.2 | 0.2 | 0.2 | 4.2 | 0.2 | 0.2 | 0.0 | 0.5 | 0.1 | 0.1 | 0.0 | 0.0 | 0.0 | 0.5 | 1.8 | 0.2 | 0.5 | 0.1 | 0.1 | 0.7 | 76.0 |
| *F. affinis* | 5.2 | 7.0 | 0.6 | 5.9 | 31.8 | 9.8 | 1.2 | 0.4 | 1.8 | 0.7 | 1.2 | 0.0 | 8.3 | 1.8 | 0.7 | 0.0 | 0.7 | 0.3 | 0.7 | 0.0 | 0.0 | 0.0 | 0.9 | 0.7 | 0.5 | 1.9 | 0.0 | 1.2 | 10.9 | 5.9 |
| *F. amabilis* | 3.1 | 5.9 | 23.7 | 2.9 | 0.3 | 1.5 | 6.5 | 0.8 | 2.6 | 0.2 | 0.6 | 0.6 | 24.4 | 0.7 | 0.3 | 0.0 | 1.6 | 2.5 | 0.0 | 0.0 | 0.2 | 0.4 | 2.2 | 2.4 | 0.0 | 0.5 | 0.0 | 0.0 | 0.1 | 16.0 |
| *F. amana* | 4.7 | 14.2 | 2.7 | 5.0 | 9.4 | 10.1 | 6.7 | 6.4 | 8.7 | 0.7 | 0.8 | 0.4 | 3.4 | 0.0 | 0.8 | 2.7 | 0.0 | 1.9 | 0.3 | 1.2 | 0.0 | 0.4 | 11.6 | 2.7 | 3.4 | 0.0 | 0.7 | 0.1 | 0.6 | 0.3 |
| *F. camtschatcensis* | 5.4 | 5.8 | 0.3 | 6.8 | 32.6 | 9.2 | 1.0 | 0.3 | 0.7 | 0.6 | 0.9 | 0.0 | 7.9 | 1.9 | 0.5 | 0.0 | 0.6 | 0.0 | 0.7 | 0.0 | 0.0 | 0.0 | 0.5 | 0.5 | 0.0 | 1.6 | 0.0 | 0.6 | 15.5 | 6.1 |
| *F. carica* | 5.1 | 14.2 | 3.3 | 13.2 | 6.5 | 12.3 | 4.2 | 1.0 | 3.4 | 0.0 | 1.3 | 0.0 | 2.4 | 0.0 | 0.0 | 0.0 | 1.9 | 0.0 | 0.0 | 0.0 | 0.0 | 4.8 | 2.3 | 2.6 | 1.3 | 2.8 | 0.8 | 0.6 | 10.7 | 5.4 |
| *F. crassifolia* | 4.7 | 11.7 | 1.3 | 4.8 | 1.5 | 6.0 | 3.5 | 1.6 | 0.0 | 0.0 | 3.0 | 0.0 | 3.5 | 0.0 | 0.0 | 0.0 | 2.0 | 2.5 | 0.0 | 0.0 | 0.0 | 0.0 | 1.2 | 1.8 | 1.8 | 0.0 | 1.2 | 0.3 | 47.7 | 0.0 |
| *F. drenovskii* | 5.2 | 7.1 | 1.0 | 5.1 | 23.4 | 7.8 | 1.6 | 0.3 | 1.0 | 1.4 | 2.0 | 0.0 | 18.1 | 2.5 | 1.3 | 0.0 | 1.6 | 0.2 | 0.0 | 0.0 | 0.0 | 0.0 | 3.1 | 0.0 | 0.0 | 2.3 | 0.0 | 0.7 | 7.4 | 6.8 |
| *F. eastwoodiae* | 7.1 | 15.2 | 2.4 | 7.6 | 6.0 | 7.8 | 6.4 | 3.3 | 7.0 | 1.0 | 1.1 | 0.3 | 11.3 | 0.0 | 0.0 | 0.0 | 2.3 | 1.2 | 0.3 | 0.8 | 0.0 | 1.0 | 7.0 | 2.4 | 0.0 | 3.3 | 0.0 | 0.2 | 2.6 | 2.4 |
| *F. eduardii* | 0.3 | 0.5 | 0.2 | 2.0 | 0.5 | 4.0 | 0.6 | 0.1 | 0.6 | 0.0 | 0.0 | 0.1 | 88.7 | 0.0 | 0.0 | 0.0 | 0.2 | 0.0 | 0.0 | 0.1 | 0.0 | 0.0 | 0.7 | 0.3 | 0.2 | 0.5 | 0.0 | 0.0 | 0.0 | 0.3 |
| *F. ehrhartii* | 3.0 | 27.3 | 3.8 | 0.9 | 0.0 | 2.8 | 2.4 | 0.4 | 2.3 | 0.0 | 3.0 | 0.0 | 17.2 | 0.0 | 1.2 | 0.0 | 0.7 | 1.5 | 0.0 | 0.0 | 0.0 | 2.1 | 1.6 | 0.9 | 0.9 | 0.9 | 0.0 | 1.6 | 21.5 | 4.1 |
| *F. elwesii* | 2.6 | 4.2 | 32.3 | 3.9 | 0.9 | 2.7 | 4.4 | 0.4 | 2.8 | 0.4 | 0.5 | 0.3 | 18.9 | 0.1 | 0.1 | 0.0 | 1.6 | 0.1 | 0.1 | 0.0 | 0.0 | 0.2 | 3.9 | 1.2 | 0.1 | 0.6 | 0.1 | 0.0 | 0.2 | 17.3 |
| *F. gentneri* | 4.2 | 10.8 | 2.1 | 4.5 | 21.2 | 7.5 | 2.1 | 0.2 | 1.6 | 0.7 | 1.1 | 0.0 | 22.7 | 2.2 | 1.8 | 0.0 | 1.0 | 0.1 | 0.9 | 0.0 | 0.4 | 0.4 | 1.1 | 0.8 | 0.2 | 1.3 | 0.0 | 0.6 | 4.2 | 6.4 |
| *F. gracilis* | 3.9 | 24.1 | 3.2 | 1.4 | 0.4 | 1.4 | 4.3 | 0.6 | 1.6 | 0.6 | 0.2 | 0.3 | 46.5 | 0.0 | 0.5 | 0.0 | 0.3 | 0.0 | 0.0 | 1.0 | 0.0 | 0.0 | 2.2 | 2.9 | 0.5 | 0.0 | 0.0 | 0.4 | 0.8 | 2.9 |
| *F. graeca* | 2.8 | 10.2 | 3.4 | 5.0 | 3.4 | 4.2 | 3.6 | 1.8 | 2.4 | 0.8 | 1.4 | 0.0 | 32.7 | 0.0 | 2.5 | 0.0 | 1.2 | 0.4 | 0.6 | 0.0 | 0.6 | 0.0 | 1.5 | 1.8 | 1.3 | 0.0 | 0.0 | 0.4 | 17.8 | 0.0 |
| *F. grandiflora* | 0.7 | 2.8 | 4.8 | 6.7 | 1.5 | 15.1 | 8.3 | 13.8 | 4.0 | 0.2 | 0.4 | 0.7 | 27.3 | 0.1 | 0.5 | 0.0 | 0.5 | 0.1 | 0.3 | 0.7 | 0.2 | 1.0 | 3.4 | 3.3 | 0.0 | 1.4 | 0.8 | 0.4 | 0.0 | 1.0 |
| *F. imperialis* | 1.8 | 15.7 | 1.0 | 2.8 | 2.3 | 12.7 | 2.1 | 0.1 | 1.4 | 0.4 | 0.2 | 0.4 | 53.1 | 0.2 | 0.4 | 0.0 | 0.2 | 0.0 | 0.0 | 0.0 | 0.2 | 0.4 | 0.6 | 0.9 | 0.1 | 0.7 | 0.0 | 0.0 | 0.5 | 1.9 |
| *F. kotschyana* | 1.7 | 32.5 | 1.1 | 3.1 | 0.4 | 2.8 | 5.5 | 6.5 | 4.6 | 0.0 | 0.0 | 0.3 | 26.7 | 0.0 | 7.8 | 0.4 | 0.1 | 0.1 | 0.0 | 0.2 | 2.7 | 0.2 | 2.3 | 0.0 | 0.7 | 0.0 | 0.1 | 0.0 | 0.0 | 0.2 |
| *F. liliacea* | 4.9 | 11.4 | 1.4 | 6.0 | 14.7 | 9.2 | 7.6 | 0.6 | 3.2 | 0.8 | 0.7 | 0.5 | 11.7 | 1.5 | 1.8 | 0.0 | 1.0 | 0.2 | 0.4 | 1.0 | 0.0 | 1.1 | 0.9 | 4.0 | 3.4 | 1.7 | 0.2 | 0.3 | 5.5 | 4.4 |
| *F. lusitanica* | 4.5 | 17.6 | 1.8 | 4.6 | 0.0 | 4.4 | 3.1 | 0.7 | 2.3 | 2.6 | 1.2 | 0.0 | 10.1 | 0.0 | 0.4 | 0.0 | 0.9 | 0.0 | 0.5 | 1.0 | 0.0 | 1.1 | 1.1 | 1.5 | 1.2 | 1.4 | 0.0 | 2.6 | 24.3 | 11.1 |
| *F. meleagris* | 2.2 | 4.7 | 1.4 | 3.7 | 0.0 | 4.8 | 3.6 | 0.5 | 0.0 | 0.3 | 0.9 | 0.0 | 65.8 | 0.6 | 0.8 | 0.0 | 0.5 | 0.0 | 0.3 | 0.6 | 0.0 | 0.3 | 1.7 | 3.0 | 0.0 | 1.6 | 0.1 | 0.2 | 0.8 | 1.7 |
| *F. meleagroides* | 6.0 | 20.8 | 1.0 | 8.0 | 18.4 | 9.9 | 3.7 | 0.5 | 1.7 | 0.9 | 1.0 | 0.1 | 3.8 | 1.7 | 2.2 | 0.0 | 0.8 | 0.0 | 0.9 | 7.0 | 0.3 | 0.9 | 0.7 | 1.2 | 0.1 | 1.2 | 0.1 | 0.3 | 2.5 | 4.0 |
| *F. michailovskyi* | 4.2 | 5.8 | 0.7 | 4.4 | 23.1 | 6.6 | 5.7 | 0.4 | 1.2 | 0.8 | 0.8 | 0.1 | 9.7 | 2.6 | 0.8 | 0.0 | 0.7 | 0.0 | 0.7 | 5.6 | 0.1 | 0.2 | 1.0 | 4.1 | 0.1 | 2.7 | 0.4 | 0.4 | 12.5 | 4.8 |
| *F. pallidiflora* | 3.7 | 5.1 | 4.9 | 5.1 | 18.2 | 7.9 | 2.2 | 0.6 | 2.5 | 0.8 | 2.9 | 0.6 | 15.2 | 2.1 | 5.5 | 1.7 | 1.0 | 0.6 | 1.8 | 0.4 | 1.4 | 0.0 | 1.3 | 0.9 | 1.0 | 1.4 | 0.8 | 0.7 | 5.4 | 4.1 |
| *F. persica* | 1.8 | 37.4 | 2.8 | 3.7 | 2.9 | 16.4 | 1.7 | 0.6 | 1.2 | 0.0 | 0.0 | 0.0 | 15.6 | 0.0 | 0.0 | 0.0 | 0.9 | 0.0 | 0.7 | 0.0 | 0.0 | 1.3 | 2.2 | 0.8 | 1.0 | 0.0 | 0.7 | 0.1 | 8.1 | 0.0 |
| *F. pontica* | 1.4 | 6.3 | 3.6 | 7.3 | 6.4 | 5.4 | 5.7 | 17.5 | 6.7 | 0.7 | 0.5 | 0.6 | 26.3 | 0.7 | 0.3 | 0.0 | 0.4 | 0.0 | 0.2 | 0.3 | 0.1 | 0.0 | 1.5 | 3.0 | 0.3 | 0.8 | 0.1 | 0.1 | 2.4 | 1.3 |
| *F. pyrenaica* | 2.5 | 2.2 | 1.0 | 6.4 | 4.2 | 3.6 | 41.4 | 0.7 | 1.3 | 0.0 | 2.3 | 0.0 | 6.3 | 0.0 | 7.6 | 0.0 | 0.0 | 0.0 | 1.7 | 0.0 | 1.6 | 2.6 | 0.0 | 0.7 | 1.1 | 0.0 | 5.5 | 0.7 | 4.4 | 2.1 |
| *F. raddeana* | 2.9 | 10.5 | 2.3 | 5.5 | 0.0 | 8.6 | 4.0 | 0.7 | 3.8 | 2.9 | 1.4 | 0.0 | 38.8 | 0.0 | 0.5 | 0.0 | 1.3 | 0.0 | 0.6 | 0.0 | 0.0 | 1.2 | 2.2 | 2.0 | 0.0 | 2.4 | 0.0 | 0.0 | 2.7 | 5.6 |
| *F. recurva* | 6.4 | 9.7 | 0.7 | 3.8 | 30.9 | 10.6 | 1.8 | 0.0 | 0.2 | 0.7 | 1.1 | 0.0 | 14.4 | 2.7 | 1.2 | 0.0 | 0.9 | 0.2 | 0.9 | 0.0 | 0.0 | 0.0 | 1.2 | 0.6 | 0.4 | 2.0 | 0.0 | 0.7 | 2.7 | 6.4 |
| *F. sewerzowii* | 3.4 | 6.4 | 1.7 | 3.8 | 17.9 | 6.6 | 1.7 | 0.2 | 0.9 | 0.9 | 1.2 | 0.6 | 31.7 | 2.0 | 1.7 | 1.2 | 0.9 | 0.0 | 0.8 | 0.2 | 1.6 | 0.0 | 0.9 | 0.7 | 0.6 | 2.1 | 0.0 | 0.3 | 2.3 | 7.6 |
| *F. stenanthera* | 3.9 | 9.9 | 0.0 | 4.2 | 0.0 | 10.9 | 3.0 | 1.3 | 3.5 | 0.8 | 1.0 | 0.0 | 3.3 | 0.0 | 0.0 | 0.0 | 1.0 | 1.4 | 0.6 | 0.0 | 0.0 | 1.4 | 1.7 | 2.6 | 3.2 | 1.6 | 0.0 | 0.6 | 38.2 | 6.0 |
| *F. stribrnyi* | 10.5 | 17.3 | 1.3 | 3.2 | 1.1 | 3.0 | 7.7 | 0.3 | 3.4 | 0.0 | 0.5 | 0.0 | 34.8 | 0.0 | 0.4 | 0.0 | 1.3 | 0.0 | 0.0 | 0.0 | 0.0 | 0.9 | 3.5 | 3.9 | 0.0 | 1.8 | 0.2 | 0.3 | 3.9 | 0.7 |
| *F. thessala* | 0.8 | 1.6 | 1.7 | 7.4 | 1.5 | 2.2 | 6.4 | 37.0 | 0.0 | 0.7 | 0.4 | 0.5 | 18.6 | 0.0 | 0.5 | 8.4 | 0.0 | 0.0 | 0.2 | 0.4 | 0.0 | 0.6 | 0.5 | 5.4 | 0.9 | 0.0 | 0.0 | 0.1 | 2.5 | 1.7 |
| *F. tubiformis* | 1.3 | 5.0 | 3.2 | 9.3 | 1.1 | 3.9 | 6.3 | 0.6 | 3.5 | 0.0 | 0.3 | 0.6 | 56.6 | 0.0 | 0.7 | 0.0 | 0.3 | 0.1 | 0.0 | 0.1 | 0.1 | 0.2 | 1.8 | 2.2 | 0.0 | 1.5 | 0.1 | 0.0 | 0.2 | 1.0 |
| *F. ussuriensis* | 2.3 | 24.4 | 4.2 | 0.5 | 0.0 | 0.8 | 15.9 | 14.9 | 0.0 | 0.4 | 0.1 | 0.1 | 22.8 | 0.0 | 0.7 | 0.0 | 0.4 | 0.0 | 0.0 | 0.0 | 0.0 | 0.4 | 3.0 | 6.1 | 0.0 | 1.1 | 0.1 | 0.5 | 1.0 | 0.3 |
| *F. uva vulpis* | 3.1 | 38.5 | 1.2 | 7.7 | 6.3 | 12.5 | 9.0 | 1.3 | 0.9 | 0.0 | 0.2 | 0.0 | 0.8 | 0.0 | 1.5 | 0.6 | 0.0 | 0.3 | 2.9 | 0.3 | 0.3 | 3.7 | 1.9 | 0.0 | 3.6 | 0.0 | 0.5 | 0.5 | 1.3 | 0.9 |
| *F. verticillata* | 2.1 | 1.1 | 0.0 | 4.8 | 1.9 | 3.2 | 1.3 | 0.3 | 0.9 | 0.0 | 0.8 | 0.0 | 6.4 | 0.0 | 0.0 | 0.7 | 0.0 | 0.0 | 0.3 | 0.4 | 0.4 | 1.0 | 0.8 | 0.5 | 1.0 | 0.0 | 0.0 | 0.2 | 56.4 | 15.4 |
| *F. whittallii* | 3.3 | 11.0 | 2.5 | 3.5 | 1.1 | 7.4 | 5.1 | 2.4 | 2.3 | 0.0 | 0.0 | 0.0 | 45.7 | 0.0 | 0.0 | 0.0 | 1.4 | 0.0 | 0.4 | 0.0 | 0.0 | 1.6 | 3.7 | 0.0 | 1.3 | 0.3 | 0.4 | 0.1 | 5.8 | 0.8 |
